# Efficient Multi-Material Volume Rendering for Realistic Visualization with Complex Transfer Functions

**DOI:** 10.3390/jimaging11060193

**Published:** 2025-06-11

**Authors:** Chunxiao Xu, Xinran Xu, Jiatian Zhang, Yiheng Cao, Lingxiao Zhao

**Affiliations:** 1 School of Biomedical Engineering, Division of Life Sciences and Medicine, University of Science and Technology of China, Hefei 230026, China; feimos@mail.ustc.edu.cn (C.X.); xuxinran@mail.ustc.edu.cn (X.X.); zjt20174025@mail.ustc.edu.cn (J.Z.); 2 Suzhou Institute of Biomedical Engineering and Technology, Chinese Academy of Sciences, Suzhou 215613, China; caoyh@sibet.ac.cn

**Keywords:** medical visualization, realistic volume rendering, volumetric accelerator, transfer function

## Abstract

Physically based realistic direct volume rendering (DVR) is a critical area of research in scientific data visualization. The prevailing realistic DVR methods are primarily rooted in outdated theories of participating media rendering and often lack comprehensive analyses of their applicability to realistic DVR scenarios. As a result, the fidelity of material representation in the rendered output is frequently limited. To address these challenges, we present a novel multi-material radiative transfer model (MM-RTM) designed for realistic DVR, grounded in recent advancements in light transport theories. Additionally, we standardize various transfer function techniques and propose five distinct forms of transfer functions along with proxy volumes. This comprehensive approach enables our DVR framework to accommodate a wide range of complex transfer function techniques, which we illustrate through several visualizations. Furthermore, to enhance sampling efficiency, we develop a new multi-hierarchical volumetric acceleration method that supports multi-level searches and volume traversal. Our volumetric accelerator also facilitates real-time structural updates when applying complex transfer functions in DVR. Our MM-RTM, the unified representation of complex transfer functions, and the acceleration structure for real-time updates are complementary components that collectively establish a comprehensive framework for realistic multi-material DVR. Evaluation from a user study indicates that the rendering results produced by our method demonstrate the most realistic effects among various publicly available state-of-the-art techniques.

## 1. Introduction

Direct volume rendering (DVR) plays an indispensable role in scientific data visualization [1,2,3], especially for the informative presentation and interpretation of medical image data [1,4]. DVR techniques [5,6] primarily evolved from simulations of light transport [7,8]. They make use of transfer functions [9] to map volumetric data values to material properties. With the development of computing technology, specifically benefitting from the significantly improved performance of Graphics Processing Units (GPUs) and the refined DVR theories, researchers have been able to explore the application of tailored DVR techniques for specified visualization tasks and effects [10,11,12].

The realism of rendering comes from simulating light transport and how light interacts with materials [13]. Some research works have verified that incorporating advanced illumination techniques in DVR can create realistic representations of materials with shadow casting and help enhance the observer’s spatial awareness and comprehension of the data [14,15]. Numerous studies have attempted to integrate various illumination algorithms in DVR to produce different shading effects and visual outcomes [15,16,17,18]. The volumetric path tracing (VPT) based on Monte Carlo (MC) estimations has been introduced in DVR to generate photorealistic rendering results [19,20,21]. VPT-based cinematic rendering techniques have been predominantly utilized in the 3D visualization of medical images, demonstrating substantial usability in the clinical diagnosis of diseases [4,22,23,24].

The radiative transfer equation (RTE) can be used to simulate the light propagation within non-refractive participating media. VPT-based methods for estimating RTE developed in recent years [25] were primarily based on the null-collision technique [26]. Some techniques are used to efficiently compute transmittance [27,28], while others are employed for global illumination sampling [29,30,31]. These methods deserve attention for their conciseness, efficiency, and analytical tractability. However, these methods are primarily applied to rendering participating media such as clouds or smoke. In contrast to participating media, which are sparse volumetric data, scientific volumetric data exhibits a higher level of detail and includes implicit surface structures. The rendering of such data requires a more accurate representation of its intrinsic details.

There are many techniques for realistic volumetric rendering, most of which involve various approximations to shading models and materials, including approximations for lighting [32], material types [33], and sampling [34]. These approximation methods result in limitations in the rendered effects, particularly in terms of the realism of lighting and shadows. Besides, although many studies advocate the use of VPT as the DVR technique, they often lack specific implementation details. Several implementations are derived from the ray-marching method used in ExposureRenderer [34], essentially serving as an approximation of VPT. In practice, using VPT for DVR without modifications usually produces visualizations with low contrast and insufficient detail. We analyze this issue and propose effective solutions to enhance the realism of VPT-based DVR.

The transfer function maps voxel values to various optical properties [9]. The diverse transfer function techniques employed by existing DVR methods complicate their integration into one system. To address this, we analyzed and unified the various transfer function techniques, proposing five primary types. We demonstrate that current transfer function techniques can be transformed into the following form: *N* (where N≥1) proxy volumes with N-dimensional transfer functions of these five types. Our classification approach enhances the utilization of multi-material radiative transfer models (MM-RTM), improving the realism of volume rendering results.

When the density within the volumetric space exhibits significant variation, a volume acceleration structure is typically employed to enhance sampling efficiency [35]. However, existing acceleration methods often encounter issues related to sampling efficiency or interactivity. Interactive DVR requires the acceleration structure to respond in real time to changes in the transfer function while ensuring that the dynamically constructed structure aligns closely with the volumetric density. To address this, a hierarchical volume acceleration structure is designed that supports empty space skips and multi-level volume traversal [13], significantly improving rendering efficiency compared to existing methods.

**Figure 1:**
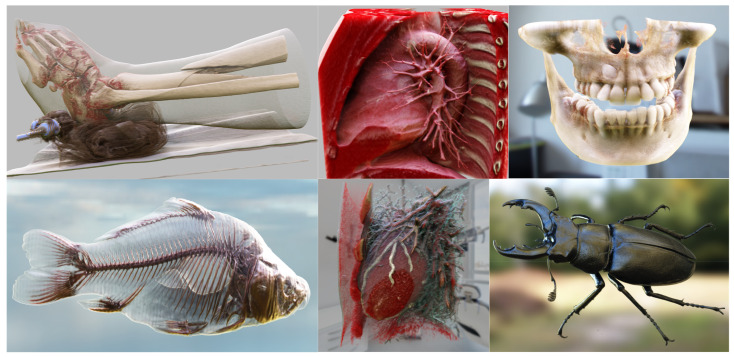
Our multi-material radiative transfer model supports physically based rendering of multiple scattering effects. It can be used to significantly enhance the visual authenticity of the volume rendering results. Our rendering results exhibit high realism, such as the subsurface scattering effect observed in teeth, the smooth surface of the beetle, and the semi-translucent appearance of muscles and blood vessels.

Our research tries to extend the RTE to support realistic DVR for physically based visualization of multiple materials. Realistic DVR results generated using our method are depicted in Figure 1; unless otherwise specified, the datasets utilized in this paper are sourced from [36,37]. Our contributions include:

- We develop a new MM-RTM that supports the VPT-based DVR of multiple materials. Our method is capable of incorporating theoretical multiple scattering effects into realistic DVR.
- We propose a novel hierarchical volume grid acceleration structure. An update mechanism is designed for this acceleration structure that ensures accurate real-time updates during user interaction. Our update method supports applications involving complex transfer functions.
- We standardized the representation of transfer functions and identified five fundamental types. We demonstrate that complex transfer functions can be expressed in these five forms.

Our MM-RTM, the unified representation of complex transfer functions, and the acceleration structure for real-time updates are complementary components that together create a comprehensive framework for realistic DVR. We validated the effectiveness of the system through two medical applications, and after evaluation by medical experts, the system is adequate for surgical planning in these two surgical scenarios. In our Supplementary Material, we provide rendering results for real-time browsing. We include scenes reconstructed using neural rendering techniques, featuring real-time browsing effects achieved with neural radiance field (NeRF) [38,39] and 3D Gaussian splatting (3DGS) [40,41].

## 2. Related Work

The DVR technique has a wide range of applications in the field of scientific data visualization. It is used to improve human perception and understanding of volumetric data. Various literature offer in-depth insight into this research domain [1,3]. Given that scientific data are usually derived from nature, visualization outcomes tend to possess a certain degree of realism or offer visual enhancements for specific regions of interest [9,11,14,42]. The realism of rendering often stems from the simulation of light transport and interactions between light and materials [13,15].

Previous studies [1,14] have demonstrated that advanced illumination can enhance people’s spatial perception of rendering results. A comprehensive overview of advanced volumetric illumination techniques in interactive DVR is provided in the survey [15]. Numerous classic rendering techniques, including precomputed radiance transfer (PRT), photon mapping (PM), and virtual point lights (VPLs), have been adopted in DVR to achieve advanced shading effects. Bauer et al. [43] introduced photon field networks, which are neural representations that facilitate interactive rendering with diminished stochastic noise. Wu et al. [44] proposed an efficient technique for volumetric neural representation. Their method performs real-time volumetric photon tracing using a newly developed sampling stream algorithm.

Advanced transfer function techniques can more effectively represent volumetric data information [9]. Various transfer function techniques use low-dimensional, high-dimensional, and even semantic information for classification. Igouchkine et al. [11] introduced a material transfer function that facilitates high-quality rendering of multi-material volumes by generating surface-like behavior at structure boundaries and volume-like behavior within the structures. With the advancement of deep learning, automated classification methods based on semantic information have emerged. Nguyen et al. [45] proposed a transfer function technique utilizing deep learning to generate soft labels for visualizing electron microscopy data with high noise levels. Engel et al. [46] employed a large network model for semantic pre-classification of volumetric data, effectively distinguishing between different tissues and organs. Li et al. [47] proposed an attention-driven visual emphasis method for the visualization of volumetric medical images, focusing on the characterization of small regions of interest (ROIs).

In recent years, research efforts on rendering of participating media have made remarkable progress in both theories and applications [25,26]. VPT-based rendering of participating media involves techniques for sampling transmittance and free paths. Unbiased transmittance estimation includes ratio tracking and residual tracking [27]. Unbiased free path sampling techniques encompass decomposition tracking [29] and weighted tracking [26], as well as spectral tracing techniques [29] and null-scattering techniques [30,31] for estimating colored media through single-path estimation. These techniques are mainly used for rendering sparse participating media such as fog, clouds, fire, and milk-like fluids.

VPT and physically based shading techniques have been widely used in DVR to generate visually realistic renderings [19,20,21]. Realistic DVR techniques primarily rely on incorporating surface material characteristics into volumetric shading [15]. Cinematic rendering approaches of medical images [4,24,48,49,50] have become a rapidly developing research direction. These techniques can be used to effectively assist doctors in understanding and analyzing pathologies. Denisova et al. [33] proposed a DVR system that accommodates multiple scattering. VPT techniques based on MC estimation often contain lots of noise during interaction. In recent years, denoising techniques for MC-based rendering have been introduced into VPT [51,52,53] to enhance the rendering quality. Taibo et al. [53] proposed an immersive 3D medical visualization system, leveraging extended linear regression denoising for real-time cinematic rendering in head-mounted display (HMD) devices. Xu et al. [54] realized real-time rendering with high quality and temporal stability in VPT-based DVR by developing a decoupling-based denoising technique. Their proposed techniques improve real-time rendering quality through various aspects, including sampling optimization and denoising, and have since been implemented in HMD devices [55]. As the preliminary work for this study, the focus of the research [54] was not on enhancing rendering effects but rather on utilizing existing methods [19,21]. In contrast, this study emphasizes improving the realism of rendering effects while also enhancing the efficiency of the accelerators used in previous methods.

## 3. Multi-Material Radiative Transfer Model

In this section we introduce our MM-RTM. We start by elucidating the fundamental RTE [25]. For a more comprehensive understanding of background principles, please refer to [25,29]. The meanings of the symbols used in the equations are provided in Table 1.

### 3.1. The Radiative Transfer Equation

When light propagates through the participating media, it engages with particles of varying densities and is absorbed by them. In addition, light also experiences attenuation caused by scattering into other directions when it propagates along the direction ω. This commonly refers to the phenomenon of out-scattering. Both absorption and out-scattering contribute to the attenuation of light. Combining these two factors yields the definition of extinction coefficient $\sigma_{t}\left(p\right)=\sigma_{a}\left(p\right)+\sigma_{s}\left(p\right)$, where p is a point in volume space. The attenuation of light propagating along the ω can be defined as:

(1) $$\begin{array}{c} dL\left(p,\omega \right)=-\sigma_{t}\left(p\right)L\left(p,\omega \right) \end{array}$$

Light from other directions may also scatter to the direction ω at point p, strengthening the light propagating along the direction ω. This refers to the phenomenon of in-scattering and can be defined as

(2) $$\begin{array}{c} dL\left(p,\omega \right)=\sigma_{s}\left(p\right)\int_{S^{2}}\rho \left(\omega_{i},p,\omega \right)L\left(p,\omega_{i}\right)d\omega_{i} \end{array}$$

If the point p in the media exhibits spontaneous emission, the emission equation can be defined as

(3) $$\begin{array}{c} dL\left(p,\omega \right)=\sigma_{a}\left(p\right)L_{e}\left(p,\omega \right) \end{array}$$

Integrating absorption, emission and scattering equations, the differential form of RTE [13] can be obtained:

(4) $$\begin{array}{cc} L_{s}\left(p,\omega \right) & :=\left(\frac{\sigma_{a}\left(p\right)}{\sigma_{t}\left(p\right)}L_{e}\left(p,\omega \right)+\frac{\sigma_{s}\left(p\right)}{\sigma_{t}\left(p\right)}\int_{S^{2}}\rho \left(\omega_{i},p,\omega \right)L\left(p,\omega_{i}\right)d\omega_{i}\right)\\ dL(p,\omega ) & =-\sigma_{t}\left(p\right)L_{s}\left(p,\omega \right) \end{array}$$

where $\frac{\sigma_{a}\left(p\right)}{\sigma_{t}\left(p\right)}$ and $\frac{\sigma_{s}\left(p\right)}{\sigma_{t}\left(p\right)}$ can be interpreted as the proportions of light absorbed and scattered when colliding with particles, respectively. The integral form of RTE can be defined as

(5) $$\begin{array}{cc} L(p,\omega )= & T_{r}\left(p_{l}\;↔\;p\right)L\left(p_{l},\omega \right)+\int_{0}^{l}T_{r}\left(p\;+\;t^{\prime }\omega \;↔\;p\right)\sigma_{t}\left(p\;+\;t^{\prime }\omega \right)L_{s}\left(p\;+\;t^{\prime }\omega ,\omega \right)dt^{\prime } \end{array}$$

where $p_{l}$ denotes the intersection of the ray r(p,−ω) with the volume bound. $T_{r}\left(p\;+\;t\omega \;↔\;p\right)$ represents the transmittance between two points p+tω and p in the volume:

(6) $$\begin{array}{c} T_{r}\left(p\;+\;t\omega \;↔\;p\right)=e^{-\int_{0}^{t}\sigma_{t}\left(p\;+\;t^{\prime }\omega \right)dt^{\prime }} \end{array}$$

The way of solving the RTE using the MC method typically involves populating the medium with virtual particles [25,26,56]. This technique is referred to as “null-scattering,” and the implementation approach can be found in [13].

### 3.2. Extension of RTE

Scientific volumetric data contains implicit surface structures, and the shading effects must effectively highlight surface reflection. Assuming the concurrent presence of four types of particles with various optical properties in the volume, i.e., absorptive particles, volumetric scattering particles, virtual particles, and surface scattering particles, the new characteristics of our MM-RTM can be modeled as follows:

- For particles exhibiting surface-scattering properties, we assume that the geometric normal is the volumetric gradient. The surface-scattering property characterizes hemispherical scattering. Incident light from the back side of the surface is fully absorbed by the surface-scattering particles.
- The absorptivity of a traditional medium can be elucidated as the absorption of light per unit distance [13]. For surface-scattering particles, absorptivity also encompasses the absorption of light energy per unit surface area.

State-of-the-art (SotA) bidirectional scattering distribution functions (BSDFs) can be modified to eliminate surface absorption and subsequently incorporated into our MM-RTM. But the heuristic “scattering absorptivity” plays a crucial role in realistic DVR. It can strongly influence the appearance of rendering results. This obviates the necessity of employing the multi-channel extinction coefficient $\sigma_{t}$ for rendering chromatic volume.

The constraints for the phase function ρ(·) and the BSDF f(·) are different, primarily attributed to BSDF’s inclusion of the albedo:

(7) $$\begin{array}{cc} \; & \int_{S^{2}}\rho \left(\omega_{i},p,\omega_{o}\right)d\omega_{o}=1\;\;\;\;\;\;\;\int_{H^{2}}f\left(\omega_{i},p,\omega_{o}\right)\left|G\left(p\right)\cdot \omega_{o}\right|d\omega_{o}\leq 1 \end{array}$$

Denote albedo at p as A(p), the BSDF is reformulated to derive the Proxy Scattering Function (PSF) $f_{s}$ as:

(8) $$\begin{array}{cc} \; & f_{s}\left(\omega_{i},p,\omega_{o}\right)=\left\{\begin{array}{cc} \frac{f\left(\omega_{i},p,\omega_{o}\right)\left|G\left(x\right)\cdot \omega_{o}\right|}{A(p)} & G\left(x\right)\cdot \omega_{o}>0\\ 0 & otherwise \end{array}\right. \end{array}$$

where G is geometry item [13]. The integral of $f_{s}\left(\omega_{i},p,\omega_{o}\right)$ over the spherical surface is equal to 1.0.

Volumes typically contain various surface scattering particles and volumetric scattering particles. For simplicity in formula derivation, we assume that only one type of volumetric scattering particle (with phase function ρ) and one type of surface-scattering particle (with PSF $f_{s}$) exist simultaneously. The probabilities associated with each particle type are governed by the probability *P*, ensuring $P_{f_{s}}\left(p\right)+P_{\rho }\left(p\right)=1$. Therefore, the RTE is defined as

(9) $$\begin{array}{cc} L_{n}\left(p,\omega \right) & =\frac{\sigma_{a}\left(p\right)}{\sigma_{\mathrm{maj}}}L_{e}\left(p,\omega \right)+\frac{\sigma_{n}\left(p\right)}{\sigma_{\mathrm{maj}}}L\left(p,\omega \right)\\ \; & +\frac{P_{\rho }\left(p\right)\sigma_{s}\left(p\right)}{\sigma_{\mathrm{maj}}}\int_{S^{2}}\rho \left(\omega_{i},p,\omega \right)L\left(p,\omega_{i}\right)d\omega_{i}+\frac{P_{f_{s}}\left(p\right)\sigma_{s}\left(p\right)}{\sigma_{\mathrm{maj}}}A\left(p\right)\int_{S^{2}}f_{s}\left(\omega_{i},p,\omega \right)L\left(p,\omega_{i}\right)d\omega_{i} \end{array}$$

$L_{e}\left(p,\omega \right)$ represents the radiance emitted from point p in the direction ω. All coefficients in Equation (9) can be multichannel and used to describe spectrally varying media.

### 3.3. Solution Operator

During the recursive sampling process for VPT using Equation (9), the calculation of illumination contributions is limited to cases where the camera ray intersects with a light source or luminous voxels. This approach was proved to be inefficient [13]. However, by employing an operator-based transformation [30,57,58], a more efficient estimation method is obtained. The scattering operator is defined as follows:

(10) $$\begin{array}{cc} \; & \left(V^{y}h\right)\left(p,\omega \right)=\sigma_{s}\left(p\right)\cdot \int_{S^{2}}y\left(\omega^{\prime },p,\omega \right)\left[\int_{0}^{z}T_{r}\left(p\;↔\;q\right)h\left(q,\omega^{\prime }\right)dt\right]d\omega^{\prime }\\ \; & \left(N_{s}h\right)\left(p,\omega \right)=\sigma_{n}\left(p\right)\int_{0}^{z}T_{r}\left(p\;↔\;q\right)h\left(q,\omega^{\prime }\right)dt \end{array}$$

where $q=p\;+\;t\omega^{\prime }$. Thus, the light transport is described using radiance equilibrium [57,58]:

(11) $$\begin{array}{cc} \; & L_{o}=\sigma_{a}\left(p\right)L_{e}+\left(c^{f_{s}}V^{f_{s}}+c^{\rho }V^{\rho }+N_{s}\right)L_{o}⟹L_{o}\left(I-(c^{f_{s}}V^{f_{s}}+c^{\rho }V^{\rho }+N_{s})\right)=\sigma_{a}\left(p\right)L_{e}\\ \; & ⟹L_{o}={\left(I-(c^{f_{s}}V^{f_{s}}+c^{\rho }V^{\rho }+N_{s})\right)}^{-1}\sigma_{a}\left(p\right)L_{e}⟹L_{o}=\sum_{k=0}^{\infty }{\left(c^{f_{s}}V^{f_{s}}+c^{\rho }V^{\rho }+N_{s}\right)}^{k}\sigma_{a}\left(p\right)L_{e} \end{array}$$

where *I* is an identity transformation, $c^{f_{s}}=P_{f_{s}}\left(p\right)A\left(p\right)$ and $c^{\rho }=P_{\rho }\left(p\right)$.

Intuitively, the expression $\left(c^{f_{s}}V^{f_{s}}+c^{\rho }V^{\rho }+Ns\right)L_{e}$ can be interpreted as the direct illumination radiance received at p, and then scattering (including null scattering) along the direction ω. In the field of DVR, $\sigma_{a}\left(p\right)L_{e}$ represents opacity-weighted emission [13]. Building upon Equation (11), the estimation of L(p,ω) can be decomposed into the sum of radiances along the direction ω reaching point p after different numbers of scattering (including null scattering) events. This decomposition delineates the light reaching the camera after scattering various numbers of times. VPT sampling represents the inverse process of light transport, where sampling rays accumulate contributions of direct illumination at the locations of each real scattering event.

### 3.4. Self-Occlusion

**Figure 2:**
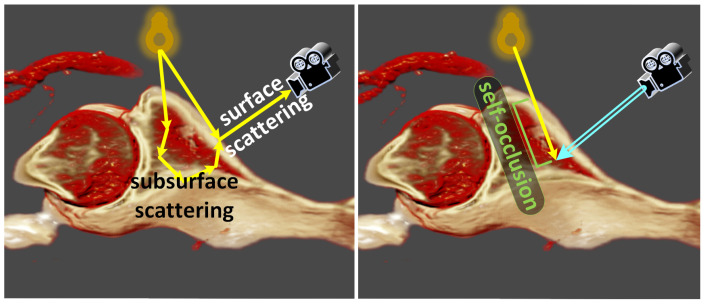
The left image depicts light scattered to the virtual camera, encompassing both surface and subsurface scattering components. However, in VPT, sampling rays may unavoidably penetrate directly into the interior of the object instead of precisely sampling the object’s implicit surface, as shown in the right image. Consequently, occluded shadow rays result in darkened renderings.

Shadow generation often encounters the phenomenon of “self-occlusion,” when using VPT in volumetric data containing implicit surface structures, as illustrated in Figure 2. For surface model rendering, light reflected from objects captured by the virtual camera includes both surface and subsurface scattering [13]. However, in DVR, sampling rays traverse implicit surfaces within the volumetric data, where the light received by sampling rays is obstructed by these implicit surfaces, resulting in darkened rendering outcomes.

**Figure 3:**
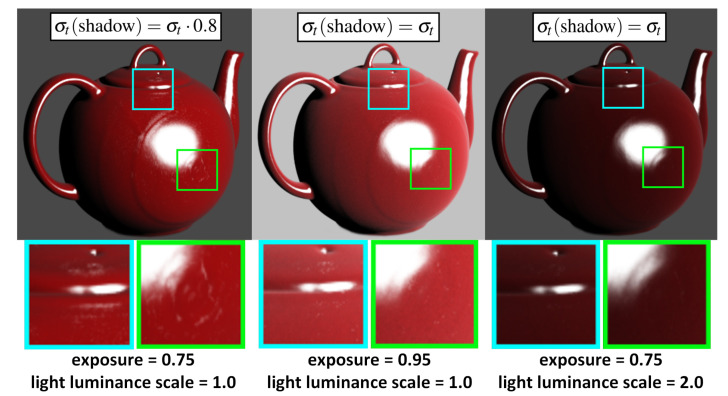
In these figures, $\sigma_{t}\;\left(\mathrm{shadow}\right)$ denotes the extinction coefficient used for sampling shadow rays. The left image depicts the rendering result obtained using our method, which involves multiplying $\sigma_{t}$ by 0.8 to obtain $\sigma_{t}\;\left(\mathrm{shadow}\right)$. The middle and right images represent sampling of free paths and shadow rays using the same extinction coefficient. The middle image enhances the overall exposure, albeit at the expense of losing shading details. Despite doubling the brightness of the light source in the right image, the rendering output remains dim.

Although increasing the brightness of the light source can enhance the brightness of the rendering results, it compromises the realism of the results, as shown in Figure 3. Some iso-surface-based DVR methods, such as the DVR method used in [11], employ recursive techniques to precisely locate surface intersections before shading computation. However, this approach diminishes sampling efficiency and compromises the visual realism of the rendering results, particularly for subsurface scattering effects. We suggest multiplying the extinction coefficient $\sigma_{t}$ of the entire volume by a factor a∈(0.75,0.85) when sampling shadow rays. It is important to note that this parameter does not affect the structural aspects of the rendering result; rather, it serves to enhance realism. The parameter *a* can be adjusted by the user. Although this approach is somewhat heuristic, it enhances the material realism of the rendering results, as shown in Figure 3.

## 4. Hierarchical Volume Grid Accelerator

Sampling efficiency can be enhanced through the use of acceleration structures. Notable accelerators include the octree [35] and the Volume Dynamic B + Tree (VDB) [59]. Some general-purpose volumetric accelerators do not facilitate real-time structure updates on GPUs [59]. A common approach to accelerating DVR involves partitioning the volumetric space into multiple grids and recursively sampling each grid using the 3D-DDA algorithm [60,61]. However, this method does not support skipping large empty areas.

Xu et al. [54] proposed an acceleration structure that combines octrees and macro cells; however, this structure does not fully leverage the sampling efficiency of volumetric hierarchies. Additionally, the design for updating this acceleration structure is relatively simplistic, as it only considers one-dimensional transfer functions. In their approach, each node stores the minimum and maximum voxel values within it, and if the transfer function changes, the maximum extinction coefficient stored at the node is updated based on the voxel range defined by these min-max values. This method is inadequate for two reasons: first, the maximum extinction coefficient calculated from the voxel range may exceed the actual maximum extinction coefficient for the node, resulting in low sampling efficiency; second, it is not applicable for more complex or multidimensional transfer functions [9].

**Figure 4:**
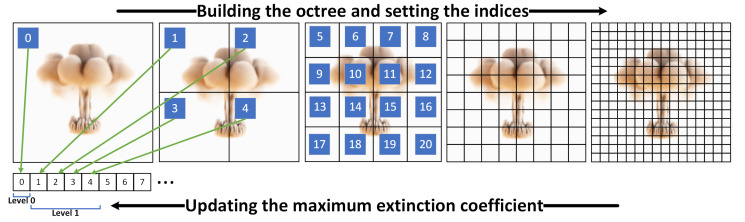
The octree is constructed in a top-down manner, starting from Level 0 and progressively building downward, with the octree information organized in a list format. Conversely, when the transfer function changes, the octree is updated in a bottom-up fashion: leaf nodes are updated first, followed by subsequent updates moving upward.

Our multi-hierarchical volumetric grid is illustrated in Figure 4. The octree is stored as a list, with each node’s information including the spatial boundary range, parent node index, indices of the eight child nodes, and the maximum extinction coefficient within that node. The leaf nodes store the index range of the volume data.

**Figure 5:**
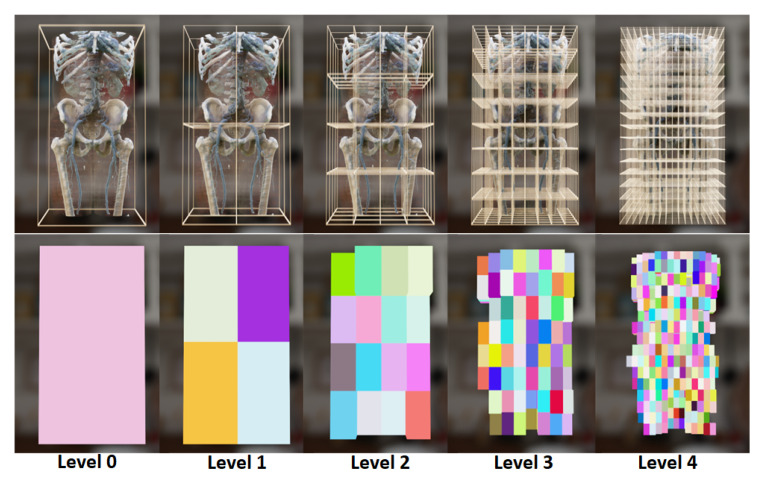
The multi-hierarchical volumetric grid. The volumetric grid is organized in the form of an octree. The first row shows the volumetric spatial range occupied by each node at every level. The second row visually distinguishes the nodes containing valid voxels at each level by displaying them in different colors.

When the transfer function changes, the acceleration structure should be updated. To achieve more accurate updates while accommodating various forms of transfer functions, a new update process is designed, as illustrated in Figure 4. First, leaf nodes are updated in parallel using CUDA, with each leaf node assigned a separate CUDA thread. Each thread traverses all voxels within the leaf node and updates the maximum extinction coefficient based on the extinction coefficient calculated from the transfer function. For levels higher than level 4, CPU parallel updating is employed, while higher levels continue to use CUDA. Nodes from Level 5 nodes are copied to CPU memory for updating the higher-level hierarchy. This update strategy enables the maximum level 7 volumetric octree to be updated in just 15 ms for $512^{3}$ resolution data, meeting real-time requirements. Figure 5 shows an example of the multi-hierarchical volumetric grid. The first row shows the volumetric spatial range of each node at different levels, while the second row visualizes the nodes containing valid voxels (those with opacity greater than 0) at each level.

To enhance sampling efficiency, we developed a new multi-hierarchical volumetric sampling method. The execution process for volumetric traversal is guided by the following principles:

- If the current node does not contain valid voxels, the query proceeds to the parent node. If the parent node also lacks valid voxels, the search continues upward. This process continues until a node is found that lacks valid voxels while its parent node contains valid voxels, at which point the sampling ray will bypass the current node.
- If the current node contains valid voxels and the maximum attenuation coefficient is less than $h_{d}$, the search will proceed upward. If the maximum attenuation coefficient exceeds $h_{u}$, the search will move downward. When the maximum attenuation coefficient is between $h_{d}$ and $h_{u}$, or if the node is at level 0 or at the leaf level, volumetric sampling will be performed. If the maximum attenuation coefficient of the current node is less than $h_{d}$, but the maximum attenuation coefficient of the parent node exceeds $h_{u}$, traversal will continue at the current node. The thresholds $h_{d}$ and $h_{l}$ are exposed as parameters to the user, allowing for multiple adjustments during practical use to observe the corresponding acceleration effects. Based on our experiments, setting $h_{d}$ to 0.2 and $h_{l}$ to 0.6 yields near-optimal traversal efficiency across multiple scenarios.
- Since the volumetric grid size is uniform across all levels, the node index of any point in any level can be directly calculated. When a ray samples within a node, it is assumed that the next node belongs to the same level; however, the search direction—upward or downward—will be determined by the maximum attenuation coefficient of the current node.

The multi-hierarchical querying mechanism we designed significantly enhances the efficiency of volume traversal. First, during 3D-DDA traversal, calculating the ray’s advance range within the current node can be relatively time-consuming. To address this, we implement a fast bounding box intersection method as described in [13], which reduces the need for extensive division calculations while still requiring a considerable amount of multiplication and addition. In contrast, the computational load associated with querying the parent node and locating child nodes is minimal. When a large area exhibits low density, volume traversal is executed directly at higher-level nodes, thereby avoiding multiple intersection calculations with the lower-level nodes. Additionally, the maximum attenuation coefficient of a node may greatly exceed that of most of its child nodes, and the downward querying mechanism improves sampling efficiency in such cases. However, it is crucial to ensure that the level of the leaf nodes is neither too low nor too high, as this can highly impact sampling efficiency. Empirically, setting the leaf node level to 6 proves to be a reasonable choice.

## 5. Multi-Dimensional Transfer Function Manager

The transfer function is utilized to map voxel values to material properties [9]. Simple transfer functions consider only low-dimensional features of the data, such as original voxel intensity values and gradient magnitudes. In contrast, complex transfer functions facilitate more targeted voxel classification based on manual user interaction. The forms of these transfer functions vary in their classification methods.

Based on our analysis, the input data for the transfer function includes both continuous data (e.g., volume intensity and gradients) and discrete data (e.g., volume labels). The output results can be categorized into two types: one corresponds to material types, such as metallic or diffuse materials, and the other corresponds to attribute values, such as opacity, roughness, and metallicity. Building on this observation and summary, we refined and integrated the transfer function techniques to ensure compatibility among different methods within the same system. Specifically, we categorize all forms of transfer functions into the following five categories: (1) coefficient curve transfer function (CCTF), (2) coefficient piecewise constant transfer function (CPCTF), (3) coefficient discrete point transfer function (CDPTF), (4) material piecewise constant transfer function (MPCTF), (5) material discrete point transfer function (MDPTF).

Each category can be applied to a proxy volume for voxel classification. A proxy volume is generated by pre-processing the original volume, which itself is also a proxy volume. Common proxy volumes include gradient magnitude volumes, opacity volumes, and mask volumes generated by segmentation. However, when multiple proxy volumes are present, conflicts may arise between the transfer function and the proxy volume. Thus, a management tool is necessary to regulate these situations, as described later.

The proxy volume represents either the original voxel values (e.g., CT volume data) or the volume data derived from the original volume data (e.g., volume gradients and labels). The proxy volume includes two forms: volumes with continuous voxel value changes, such as gradient magnitude volumes [34] and soft-labeled volumes [45] obtained through segmentation; and volumes with discrete voxel values, such as hard-labeled volumes derived from semantic segmentation [46]. Different classification methods should be applied to proxy volumes with continuous changes and those with discrete values. Specifically, volumes represented in discrete form should utilize nearest neighbor interpolation during sampling to obtain current voxel values, rather than linear interpolation, to avoid incorrect and undefined intermediate values [1,46].

**Figure 6:**
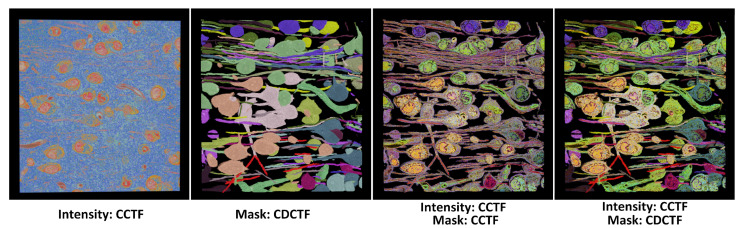
Visualization of cell volume generated via electron microscopy. The first two images from the left illustrate the classification effects on the original volume and the mask volume using a one-dimensional transfer function. The distinction between the third and fourth images lies in the different transfer functions applied to the mask volume. Notably, using a CDCTF for the discrete value mask yields a clearer delineation.

**Figure 7:**
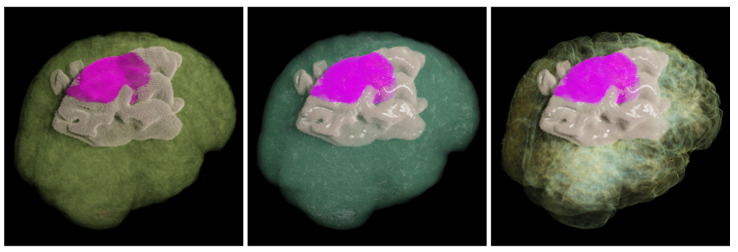
The visualization of brain tumors and tissue edema is presented. The left and middle images employ a two-dimensional transfer function, applying the same type of transfer function to both the original volume and the mask volume, resulting in the target material coefficients through their multiplication. In contrast, the right image utilizes different material types for different mask values and applies the CCTF to further classify the coefficients for each material type.

The coefficient transfer function assumes that the material of the controlled proxy volume is unique, but material properties such as roughness and reflectance are variable. This type of transfer function solely adjusts the material coefficients [34]. The relationship between the coefficient values and the voxel values of the proxy volume can take three forms: curve, piecewise constant, and discrete. In contrast, the material transfer function does not assume the uniqueness of the material in the controlled proxy volume. Since material types are discrete quantities, without intermediate transitions like coefficient values, the material transfer function is limited to either piecewise constant or discrete forms. For instance, in [33], the transfer function’s inputs include volumetric opacity values, assigning a glass material to areas of low opacity and a metallic material to areas of high opacity. This can be implemented by generating an opacity proxy volume and then applying an MPCTF to it. Another example of material transfer functions [11], which assign different discrete material types based on mask values, facilitate rendering by recursively identifying the boundaries between different materials. The effects of various transfer functions are illustrated in Figure 6 and Figure 7.

When multiple transfer function types are applied to a single volumetric dataset, their compatibility must be considered. For instance, in VTK [62], multi-dimensional transfer functions compute target coefficients by multiplying the coefficient calculated in each dimension. In cases of conflict between two types, a priority must be established. For example, in DVR of segmented results, if the original volume employs a CCTF and the mask volume uses a MDPTF, the classification method can be set to prioritize MDPTF when the opacity controlled by MDPTF exceeds zero; otherwise, CCTF is used as the classification method. As shown in the right image in Figure 7, classification using the material transfer function and coefficient transfer function simultaneously yields a visualization with significantly greater detail compared to simple coefficient multiplication.

Our transfer function technique is defined as comprising three steps: first, the generation of proxy volumes; second, the application of five types of transfer functions to assign material properties to these proxy volumes; and third, the merging of the material properties obtained from each proxy. Various complex transfer function techniques in [9], including the use of semantic segmentation for pre-processing volumetric data, can be categorized under the first step—generating proxy volumes.

**Figure 8:**
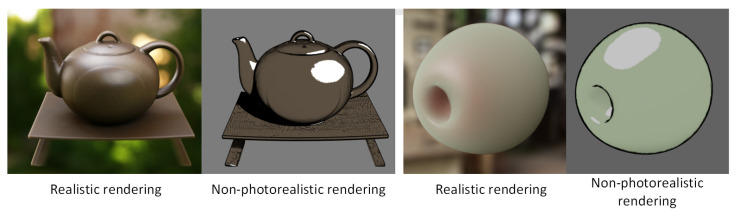
The realistic and illustrative non-photorealistic rendering effects obtained using our renderer are presented. The non-photorealistic rendering employs a two-dimensional transfer function, where the first dimension controls the material’s albedo and opacity, while the second dimension governs the shading transition.

The various combinations of transfer functions are managed by the transfer function manager. Globally, the transfer function manager takes the spatial position as input and outputs the optical properties of that position. The manager can automatically access proxy volume and execute different transfer processes based on the current user configuration. As we have noted, multiple transfer functions can be integrated into our system, requiring only minimal extensions, such as illustrative stylized rendering, as shown in Figure 8.

## 6. Implementation and Evaluations

In this section, we first present some implementation details of the renderer, followed by an evaluation of our techniques. While we have already included some comparisons of various effects in the Section 5, our evaluation encompasses three additional aspects: First, we validate the effectiveness of our system in surgical planning through two medical applications. Our system is compared with several SotA realistic volume rendering techniques; second, we conduct a user study to assess the realism of renderings produced by different photorealistic volumetric rendering algorithms; third, the sampling efficiency of our acceleration structure is compared with that of conventional acceleration structures; fourth, we tested the impact of different channel numbers of extinction coefficient on the rendering results.

### 6.1. Implementation Details

We implemented a physically-based volume renderer, with the rendering pipeline executed on CUDA. Compared to the existing open-source photorealistic volume renderer [34], our approach features several key improvements: (1) The ExposureRenderer is based on ray marching sampling techniques. We developed a VPT-based renderer using the MM-RTM proposed in this paper, which supports both single-channel and multi-channel extinction coefficients. (2) Due to the limited interpolation capabilities of CUDA for 16-bit integer textures, we opted to use 32-bit floating-point textures instead. Our renderer supports the simultaneous loading of four proxy volumes. That is, the volume data are generated as float4 -type textures. (3) The multi-hierarchical volumetric acceleration structure proposed in this paper is implemented.

Experimental hardware. All experiments were conducted on a PC workstation equipped with an Intel i7-12700KF CPU, 32 GB of RAM, and an NVIDIA GeForce RTX 4070 Ti GPU boasting 12 GB of video memory.

### 6.2. Comparison with the SotA in Surgical Planning

The most important application scenario for realistic DVR is medical image visualization, where the rendered results need to be as beneficial as possible for medical use. In this section, the advantages of our system over SotA methods in preoperative surgical planning are demonstrated. While numerous approximation and acceleration techniques have been recently developed for DVR [43,44,51,52], our comparison primarily focuses on the rendering technology. Technologies that are not compared are mainly designed for real-time acceleration rather than enhancing visualization quality; thus, they are not directly compared. The proposed system also implements schemes for real-time browsing: Supplementary Materials include scenes reconstructed using neural rendering techniques, demonstrating real-time browsing effects achieved with NeRF and 3DGS technologies.

For comparative purposes, the following SotA methods/papers were selected. We sought to reproduce their reported rendering results with high fidelity. (1) *VPL-DVR* [32] approximates global illumination by computing the direct illumination and single scattering from a set of virtual light sources. It efficiently handles transfer function and volume density updates by recomputing the contribution of these virtual lights and progressively updating their volumetric shadow maps and locations. (2) *RMSS* [21] (ray-marching-based single-scattering) is an optimized, physically based DVR technique developed based on the open-source ExposureRenderer [34] to enhance the efficiency of environment light sampling. ExposureRenderer employs the ray-marching technique for free path sampling in volume space during its implementation. (3) $AR^{2}T$ [33] represents a physically-based DVR technique supporting multiple scattering. In $AR^{2}T$, four material types are defined, and the material is determined based on the volumetric density at the current position. Dielectric material treatment is applied when the density falls below a threshold $t_{0}$, while metallic material treatment is applied when the density exceeds a threshold $t_{1}$.

**Figure 9:**
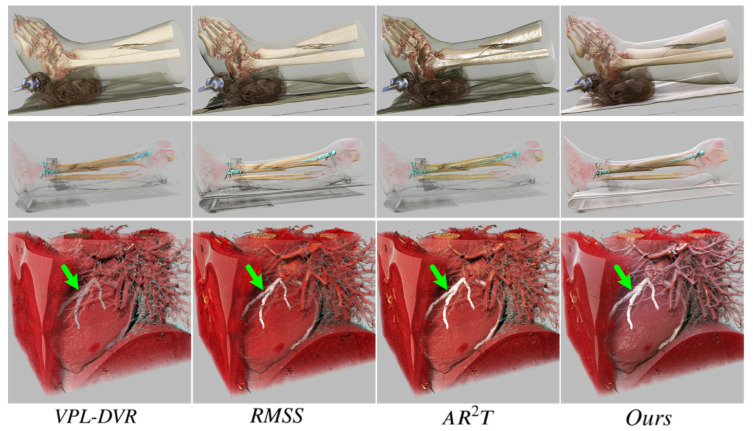
The visualization of CT data scans for patients undergoing fracture repair surgery and coronary artery stent surgery is shown. The first and second rows display the preoperative and postoperative visualizations for the fracture surgery, respectively. The third row illustrates the preoperative visualization for the stent surgery, with green arrows indicating the site of arterial stenosis.

This study explores the advantages of our realistic DVR framework in surgical planning, including two application scenarios: (1) CT imaging plays a crucial role in both the diagnosis and treatment of fractures. It provides detailed multi-layered and multi-angular images, helping physicians to accurately identify the location, fracture patterns, and fragment conditions. Three-dimensional visualization of the fracture site is an essential component of surgical planning. (2) Coronary artery stenosis, often caused by atherosclerosis, can lead to myocardial ischemia and even myocardial infarction. During interventional surgery, physicians first use a balloon to dilate the artery and then implant a stent to maintain vessel patency, ensuring adequate blood flow to the myocardium. Three-dimensional visualization of coronary angiography data is an indispensable part of surgical planning. The results obtained using different visualization methods for two distinct scenarios are shown in Figure 9.

We invited 8 clinical medical experts to evaluate the visualization results of different methods in this user study. Three evaluation criteria were established to ensure fair assessments, as outlined in Table 2. After a ten-minute evaluation of rendering results, participants rated their experiences on a scale from 1 to 5 (a higher score indicates better performance). Our statistical analysis employed one-way analysis of variance (ANOVA) to test the null hypothesis of equal correctness means across techniques. A *p*-value of ≤0.05 indicated a statistically significant difference in the scoring results between different methods.

**Figure 10:**
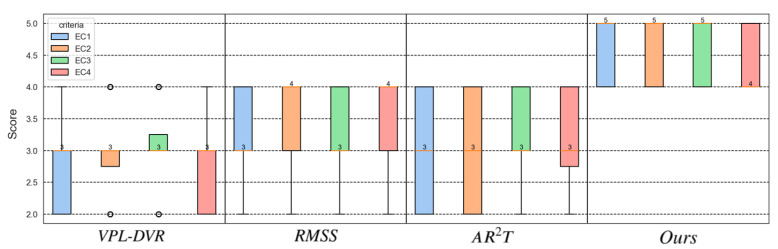
Visualization of the user ratings evaluated for the four given criteria. The boxes and whiskers display the minimum, maximum, and median (as a red horizontal line within the box).

The scoring statistical results of the user study are presented in Figure 10. The scoring results indicate that medical experts unanimously consider our method to be the most ideal visualization solution. They confirmed that our approach is sufficiently effective for application in surgical planning. The experts consistently rated our method highly and expressed a strong willingness to use it for future surgeries. While realistic rendering technologies may not enable capabilities beyond those of traditional rendering techniques, their enhanced realism and rendering details significantly improve doctors’ comfort and spatial awareness during observation. Some experts noted that higher levels of realism in the visualization make them feel more at ease, find the visuals more aesthetically pleasing, and enable them to focus more on diagnosing and planning around the lesion. To sum up, the method proposed in this paper demonstrates superior applicability for surgical planning compared to existing methods.

The differences in realism among various visualization methods will be experimentally evaluated and discussed in the next section. It is important to note that the transfer function management system presented in this paper plays a crucial role in integrating various types of volume labels and enhanced information into the rendering results. In the case of coronary angiography visualization, a two-dimensional transfer function is applied, with the first dimension being the CCTF and the second dimension the MDPTF. The coronary arteries are segmented and assigned a highlighted material type using the MDPTF. To ensure fairness, all rendering techniques employed this transfer function approach. Furthermore, a detailed comparison of the differences in rendering techniques and their effects will be provided in Section 6.3.

### 6.3. Comparison of Realistic Effects

**Figure 11:**
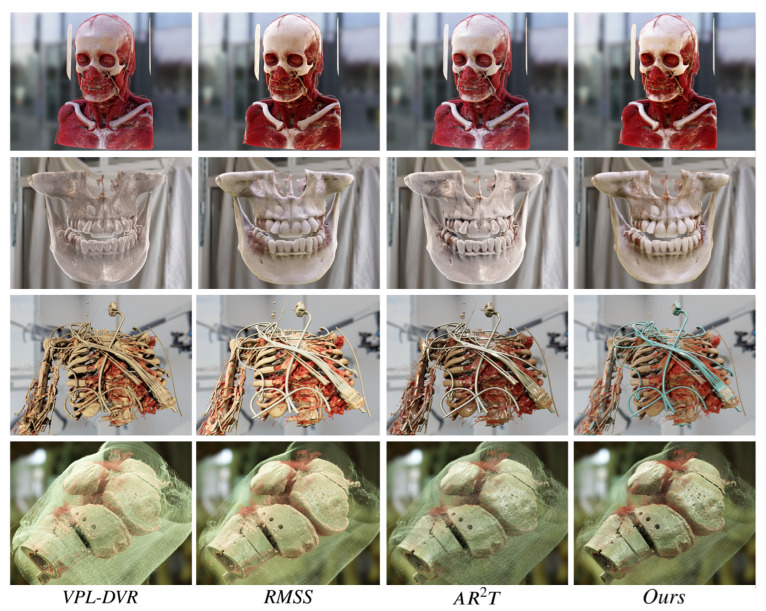
Visual comparison of realistic DVR results generated using various state-of-the-art techniques and our method.

In the user study comparing the realism of different methods, 19 participants (8 females and 11 males) aged between 23 and 30 years were recruited. Among these individuals, 12 had taken courses in medical visualization and had some familiarity with realistic DVR. Before the user study, we provided a brief overview of realistic DVR techniques to the participants. The rendering results were presented to each participant in a randomly shuffled order to minimize bias. Participants were able to freely switch between these four DVR methods and four volumetric datasets, as shown in Figure 11. High dynamic range (HDR) panorama environment maps were employed as light sources to enhance the material realism of the rendering results [10,13].

Three evaluation criteria are as outlined in Table 3. Besides evaluating the realism of rendering results, we also assessed the integration of the rendering results with the environment. To ensure the presence of rating differentiation, we encouraged users to distinguish their ratings for the results generated using different DVR methods. After a ten-minute evaluation of rendering results, participants rated their experiences on a scale from 1 to 5.

**Figure 12:**
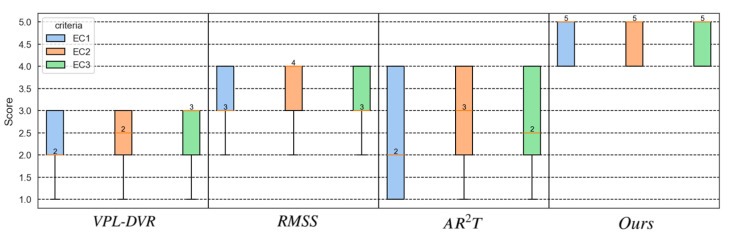
Visualization of the user ratings evaluated for the three given criteria. The boxes and whiskers display the minimum, maximum, first, and median (as a red horizontal line within the box).

The scoring statistical results of the user study are presented in Figure 12. The ANOVA test revealed significant differences among the various realistic DVR methods (*p*-value <0.001), as assessed by different criteria in Table 4. As shown in Figure 12, our realistic DVR results outperform those of other state-of-the-art DVR methods on all four datasets. Most users indicated that our rendering results significantly exhibit higher realism compared to other methods. To ensure consistency with the original rendering results of those referenced papers, we also asked users to refer to the rendered images presented in the literature of these studies [21,32,33], and users consistently affirmed the higher material realism and expressiveness of rendering results generated using our method.

The *VPL-DVR* [32] stands out as a significant technical solution based on approximate DVR global illumination. Numerous approximate global illumination approaches used in DVR can be found in [15]. Approximation methods typically involve a trade-off between rendering quality and interactive speed. The ExposureRenderer by Kroes et al. [34] is considered the forerunner of physically based realistic DVR research, exerting an important inspiration on subsequent studies in realistic DVR. The *RMSS* [21] primarily focuses on optimizing environment light sampling efficiency, sharing a similar DVR framework with the ExposureRenderer. The $AR^{2}T$ [33], a comprehensive realistic DVR solution supporting multiple scattering, exhibits limitations in its simplistic approach to material models, thereby restricting the portrayal of complex material effects.

Instead of simply combining existing techniques, we constructed a comprehensive MM-RTM. Our model offers multiple support for both surface scattering and volumetric scattering, resulting in more realistic material effects. Furthermore, we analyzed common issues in VPT-based realistic DVR, such as self-occlusion and low rendering detail, and provided corresponding solutions to address these problems. Our rendering results exhibit superior material realism, as shown in Figure 11. For instance, the metallic texture in engine, the subsurface scattering effect in tooth and bone, and the light and shadow details on the rendered results. High material realism is also beneficial for users’ comprehension and spatial perception of volume data.

### 6.4. Comparison of Accelerators

In this section, we evaluate the construction speed of different volumetric acceleration structures and their sampling efficiency. In addition to enhancing sampling performance, we introduce innovations that enable real-time updates of our acceleration structure, even for DVR with complex transfer functions. We compare various volumetric acceleration methods, including macro-cell acceleration, the Residency Octree [63], and our proposed multi-hierarchical acceleration structure. Additionally, since our accelerator allows for the configuration of different node levels, the update and sampling speeds of leaf nodes at various levels are analyzed.

Some advanced volumetric accelerators, such as SparseLeap [64], effectively skip empty space but struggle to compute compact upper bounds for extinction coefficients within each effective sampling distance. This technology is primarily applied to ray-marching-based DVR algorithms and is less suited for VPT, so we do not include it in our comparisons.

Comparisons were conducted from three aspects: (1) the time required to update the accelerator structure; (2) the time consumed to locate the nearest and farthest valid voxel positions along a ray from the camera; and (3) the time taken for global illumination estimation at 10 samples per pixel (spp). The two volumetric datasets we selected for testing are *spathorhynchus* (resolution 1024×1024×750) and *stag_beetle* (resolution 832×832×494) [36]. The test results are presented in Table 5.

As shown in Table 5, our acceleration structure demonstrates a significant advantage in real-time interaction with transfer functions. In terms of sampling efficiency, the Residency Octree [63] performs similarly to our method, which also employs a multi-hierarchical structure. However, the Residency Octree is designed for out-of-core rendering of large datasets and does not support real-time updates. In contrast, we store the octree as a sequential list, simplifying design and implementation while enabling real-time updates. Our method, being hierarchical, also supports out-of-core rendering, as illustrated in Figure 6.

### 6.5. Configurations of Different Channel Numbers

When simulating light transport through colored participating media, multi-channel $\sigma_{t}$ is commonly used [13,25]. However, employing multi-channel albedo with single-channel $\sigma_{t}$ can also generate a colored rendering effect. We will demonstrate that employing a single-channel $\sigma_{t}$ can yield rendering results indistinguishable from those obtained with multi-channel $\sigma_{t}$. Furthermore, the use of a single-channel $\sigma_{t}$ can present less noise than using multi-channel $\sigma_{t}$ during the interaction.

**Figure 13:**
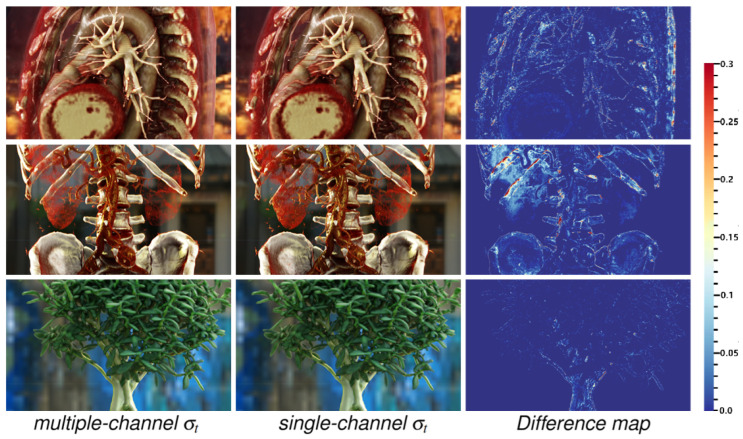
The rendering results are compared across different extinction coefficient $\sigma_{t}$ channel configurations. The difference maps illustrate the acceptable absolute value discrepancies between the rendering results of single-channel configuration and multi-channel configuration.

In Figure 13, it is observed that by adjusting the transfer function, employing a single-channel configuration yields results that are difficult to discern from those of the multi-channel configuration by the naked eye. The difference maps in Figure 13 are used to show the subtle differences between the rendering results of these two configurations.

**Figure 14:**
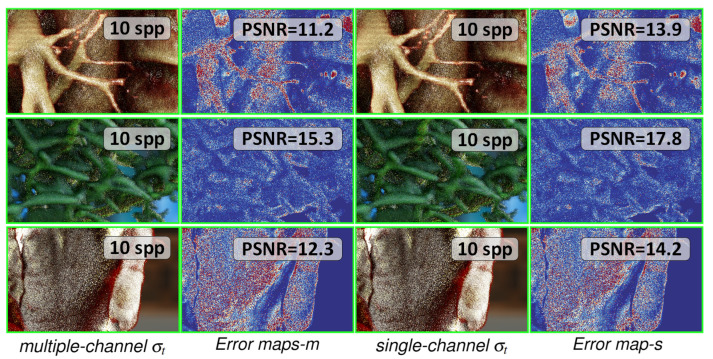
In the multi-channel version, *Error maps-m* denotes the discrepancy between rendering results with 10 spp and the reference results rendered with 4096 spp. *Error map-s* denotes the error in the single-channel rendition. A higher PSNR indicates lower image noise levels.

The rendering noise levels across the two different channel configurations of $\sigma_{t}$ were examined, as depicted in Figure 14. Single-channel configurations often exhibit lower noise levels when producing rendering effects similar to multiple-channel configurations. Therefore, we suggest that employing a single-channel $\sigma_{t}$ with multi-channel albedo is a rational and effective choice in realistic DVR settings.

## 7. Discussion

This paper presents a framework for realistic rendering of scientific volumetric data. First, we observe that many related studies still rely on early DVR techniques, and several approaches have made various approximations or imposed limitations on material and shading in DVR. As a result, the rendered effects in many studies lack realism and material expressiveness. To address this issue, we model the light transfer in multi-material volumetric data and analyze the self-occlusion phenomenon that occurs in realistic DVR. We also demonstrate, in several scenarios, that our method offers superior rendering realism compared to existing approaches.

Although we do not impose restrictions on material types, selecting the most appropriate set of materials for the current rendered object requires extensive experience and multiple iterations. The results can vary significantly depending on the individual adjusting the settings. To some extent, the process of adjusting appearance resembles artistic creation, where individuals with a deeper understanding of volumetric rendering principles and more experience in tuning parameters are more likely to achieve the desired rendering effect. The challenge of making the adjustment of rendering effects more theoretical and standardized remains an open issue.

We also integrate various transfer function techniques, managing them through a unified architecture. The transfer function manager provides a consistent interface: it takes the input of spatial coordinates and outputs material properties. The advantage of this approach is that, regardless of the complexity of the transfer functions, they remain decoupled from other components of the rendering engine. For example, even when new transfer function features are added, there is no need to modify the accelerator’s implementation code.

Although we designed the transfer function manager as a unified architecture, users still need to carefully select the appropriate transfer function configurations for different applications. If the system could integrate various preprocessing tools and select, execute, and combine modules based on natural language input from the user, it would greatly enhance the convenience of visualization.

## 8. Conclusions and Future Work

**Figure 15:**
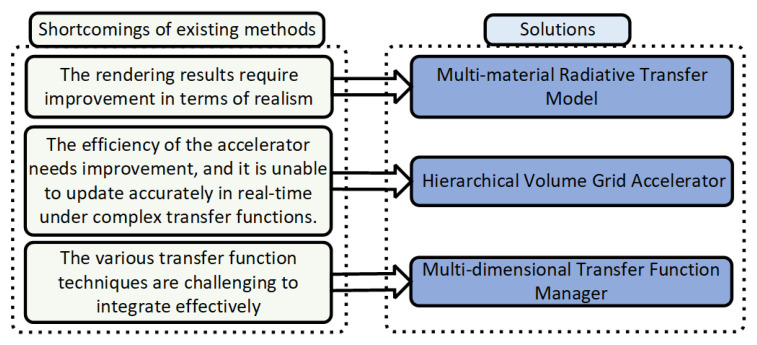
An overview of the limitations of existing methods and the solutions we propose.

In this paper, we present a photorealistic rendering framework for direct volume visualization of scientific volumetric data. As shown in Figure 15, we propose several optimization techniques aimed at addressing the issues of inadequate rendering quality in current methods.

Our primary focus is on enhancing the material realism and spatial hierarchy of the rendered results. To achieve this, we improve the state-of-the-art null-scattering technique for rendering participating media, such as clouds and smoke, enabling our volume rendering model to reveal the surface structures implicit in the volume data. Additionally, we analyze the challenges in photorealistic volume rendering and propose solutions to address these issues. Our method was compared with existing state-of-the-art realistic DVR techniques. The result showed that our method generates the most realistic effects compared to these techniques.

Furthermore, certain structures of interest require more advanced feature extraction techniques to differentiate them from other structures. To this end, we standardize various transfer function techniques and introduce five distinct forms of transfer functions along with proxy volumes, allowing our DVR framework to accommodate a variety of complex transfer function techniques effectively. This allows our rendering engine to be applied to a wide range of visualization tasks.

We also enhanced the volume accelerator structure to improve the efficiency of volume space traversal in the context of volumetric path tracing. When the render appearance changes during transfer function interaction, our accelerator structure can update in real time.

The proposed multi-material radiative transfer model, the unified form of complex transfer functions, and the acceleration structure are complementary components that together form a comprehensive framework for realistic multi-material DVR.

There are several aspects of our work that require further improvement:

(1) During interactions, we discovered that achieving high realism also depends on the proper setup of light sources. Some HDR environment maps used as illumination sources may not result in a high level of material realism in DVR. Transfer function adjustments are also necessary to obtain the desired rendering appearance under different light sources. In future work, we will conduct in-depth research to explore the relationship between light source settings and the realism of rendering results. We will also try to innovate how the transfer function can be more conveniently and intuitively adjusted to obtain desirably optimized rendering results.

(2) Our accelerator structure is designed for static volumetric data. While the appearance of the rendering result can be adjusted using transfer functions, the data itself remains unchanged. For dynamic volumetric data, such as the motion during the cardiac and respiratory cycles, more efficient data loading and updating methods need to be developed.

(3) Adjusting transfer functions requires extensive prior knowledge, particularly when dealing with complex multidimensional transfer functions, which makes the process even more challenging. In our medical application exploration, both healthcare professionals and we need to make repeated adjustments. Transfer functions can be represented using structured parameters, which can be generated and adjusted through large language models. The generated images can also be evaluated using multimodal large language models. Future research will focus on how to use large language models to intelligently generate the desired rendering effects for users, which is a crucial area of investigation.

## Figures and Tables

**Table 1:** Notations of the RTE.

Symbol	Description
p	a point in volume space
r(p,ω)	a ray, emitted from point p in the direction of ω
L(p,ω)	light from the point p to the direction ω
$\sigma_{a}\left(p\right)$	absorption coefficient at the point p
$\sigma_{s}\left(p\right)$	scattering coefficient at the point p
$\sigma_{t}\left(p\right)$	extinction coefficient at the point p
$\sigma_{n}\left(p\right)$	null-scattering coefficient at the point p
$\sigma_{\mathrm{maj}}$	the “majorant” constant, $\sigma_{\mathrm{maj}}=\sigma_{t}\left(p\right)+\sigma_{n}\left(p\right)$
$\int_{S^{2}}$	spherical integral
$\int_{H^{2}}$	the integral over the hemisphere
ρ(·)	the phase function
f(·)	the BSDF
$L_{e}\left(p,\omega \right)$	radiance emitted to ω from p
$T_{r}\left(p_{1}\;↔\;p_{2}\right)$	light transmittance between $p_{1}$ and $p_{2}$
G(p)	the gradient at the point p
A(p)	the surface scattering albedo coefficient at p
S(p)	the spherical scattering albedo coefficient at p

**Table 2:** The evaluation criteria for helping participants assess the applicability of different methods in surgical planning.

	Evaluation Criteria
*EC1*	the visualization results’ ability to support surgical planning tasks.
*EC2*	the effectiveness of the visualization in presenting three-dimensional spatial information.
*EC3*	the ability of the visualization to help doctors quickly locate the lesion.
*EC4*	the willingness to adopt this technology in surgical planning.

**Table 3:** The evaluation criteria for helping participants objectively and comprehensively evaluate different realistic DVR methods.

	Evaluation Criteria
*EC1*	The material appearance realism of rendering result.
*EC2*	Beneficial for the perception of spatial structure.
*EC3*	Integration with the background environment.

**Table 4:** The quantitative comparison of different realistic DVR methods. Apart from the *F*-value and *p*-value, each value represents the mean/standard deviation.

Method	*EC1*	*EC2*	*EC3*
*VPL-DVR* [32]	2.25/0.80	2.87/0.69	2.75/0.76
*RMSS* [21]	3.12/0.83	3.87/0.81	3.24/0.82
$AR^{2}T$ [33]	2.32/1.17	2.97/1.06	2.44/1.01
*Ours*	4.62/0.48	4.78/0.49	4.46/0.49
*F*-value	174.25	168.53	139.48
*p*-value	<0.001	<0.001	<0.001

**Table 5:** The time consumed with various configurations of accelerators while performing three tasks, measured in milliseconds (ms).

*spathorhynchus* dataset			
	Update	Locate Nearest	GI
No	0	8.23	121.71
Macrocell	7.42	7.65	84.51
Residency	135.53	5.37	64.82
Ours-6	11.14	5.62	60.63
Ours-7	13.68	6.59	72.90
*stag_beetle* dataset			
	Update	Locate Nearest	GI
No	0	8.15	105.57
Macrocell	7.16	6.94	78.73
Residency	122.63	5.17	58.15
Ours-6	10.31	5.22	59.26
Ours-7	12.45	6.12	68.14

## Data Availability

The data used in this paper, except for those in Section 6.2, are all sourced from open datasets https://www.osirix-viewer.com/ (accessed on 10 March 2020) and https://digimorph.org/ (accessed on 1 October 2024).

## Supplementary Materials

The following supporting information can be downloaded at: https://huggingface.co/datasets/feimos32/3DGS-Medical-ESWA (accessed on 6 May 2025). NeRF and 3DGS Data.
